# Allele-specific antibodies to *Plasmodium vivax* merozoite surface protein-1: prevalence and inverse relationship to haemoglobin levels during infection

**DOI:** 10.1186/s12936-016-1612-z

**Published:** 2016-11-16

**Authors:** Nuno Sepúlveda, Cristiane Guimarães Morais, Luiza Carvalho Mourão, Matheus França Freire, Cor Jesus F. Fontes, Marcus Vinícius G. Lacerda, Chris J. Drakeley, Érika Martins Braga

**Affiliations:** 1 Department of Immunology and Infection, London School of Hygiene & Tropical Medicine, London, UK; 2 Centre for Statistics and Applications of University of Lisbon, Lisbon, Portugal; 3 Departamento de Parasitologia, Instituto de Ciências Biológicas, Universidade Federal de Minas Gerais, Belo Horizonte, Brazil; 4 Departamento de Clínica Médica, Hospital Universitário Júlio Müller, Universidade Federal de Mato Grosso, Cuiabá, Brazil; 5 Fundação de Medicina Tropical Dr. Heitor Vieira Dourado, Manaus, Brazil

**Keywords:** *Plasmodium vivax*, Antibodies, MSP-1, Polymorphism, Anaemia

## Abstract

**Background:**

Antigenic polymorphisms are considered as one of the main strategies employed by malaria parasites to escape from the host immune responses after infections. Merozoite surface protein-1 (MSP-1) of *Plasmodium vivax,* a promising vaccine candidate, is a highly polymorphic protein whose immune recognition is not well understood.

**Methods and results:**

The IgG responses to conserved (MSP-1_19_) and polymorphic (block 2 and block 10) epitopes of PvMSP-1 were evaluated in 141 *P. vivax* infected patients. Ten recombinant proteins corresponding to block 2 (variants BR07, BP29, BP39, BP30, BEL) and block 10 (BR07, BP29, BP39, BP01, BP13) often observed in Brazilian *P. vivax* isolates were assessed by ELISA in order to determine levels of specific antibodies and their respective seroprevalence. The magnitude and the frequency of variant-specific responses were very low, except for BR07 variant (>40%), which was the predominant haplotype as revealed by block 10 PvMSP-1 gene sequencing. By contrast, 89% of patients had IgG against the C-terminal conserved domain (PvMSP-1_19_), confirming the high antigenicity of this protein. Using multiple linear and logistic regression models, there was evidence for a negative association between levels of haemoglobin and several IgG antibodies against block 2 variant antigens, with the strongest association being observed for BP39 allelic version. This variant was also found to increase the odds of anaemia in these patients.

**Conclusions:**

These findings may have implications for vaccine development and represent an important step towards a better understanding of the polymorphic PvMSP-1 domain as potential targets of vaccine development. These data highlight the importance of extending the study of these polymorphic epitopes of PvMSP-1 to different epidemiological settings.

**Electronic supplementary material:**

The online version of this article (doi:10.1186/s12936-016-1612-z) contains supplementary material, which is available to authorized users.

## Background

*Plasmodium vivax* represents a challenge to the public health system in the Americas with approximately 80 million people being exposed to this malaria agent and about 300,000 clinical cases registered in 2013 [1]. Although the total number of confirmed malaria cases and deaths in the region has been decreased in the last decades, *P. vivax* infections in Brazil still account for 42% of all cases and half of the deaths due to malaria registered in the Americas [1]. Several potential challenges may impact the elimination efforts in Brazil where *P. vivax* accounts for more than 80% of diagnosed malarial infections [2] and cases of severe disease due to this species has been reported in the Amazon endemic region [3–5]. Therefore, the development of an effective malaria vaccine is likely to contribute to a reduction of the disease burden in endemic populations. Notwithstanding the public health impact caused by *P. vivax*, only a few vaccine candidates have been tested in clinical trials due to an unclear protective role of the potential vaccine targets (reviewed in [6–8]).

Several studies have been performed in order to identify *P. vivax* antigens as putative targets for vaccine development. The PvMSP-1, a 200 kDa protein highly expressed on the surface of merozoites, is one of the best-characterized antigens. This protein contains six highly polymorphic domains flanked by inter and intra-specific conserved sequences [9]. Past studies on naturally acquired immune responses against PvMSP-1 variable regions showed several limitations. The most important is the focus of the analysis on recombinant proteins representing only one single version of the N-terminus of the protein. This is illustrated with examples: only two cohort studies in Brazil were able to show a reduced risk of infection and clinical protection associated to N-terminal PvMSP1-specific antibodies [10, 11]. By contrast, a similar association between antibody responses to N-terminus of PvMSP-1 and either *P. vivax* infection or asymptomatic status was not observed in another study conducted in Brazil and Papua New Guinea [12]. This lack of consistency calls for an extended analysis where a broader representation of the PvMSP-1 repertoire must be included in further studies. With this in mind, Bastos and colleagues used a panel of different variants comprising three polymorphic PvMSP-1 domains (blocks 2, 6 and 10). They showed evidence for a positive correlation between cumulative exposure to malaria and presence and levels of IgG antibodies to many PvMSP-1 variant antigens [13].

Whilst there is strong evidence showing that anti-PvMSP-1 antibodies are associated with cumulative exposure rather protection against vivax malaria, the relative contribution of different regions of the molecule inducing naturally acquired antibodies remains unknown. Therefore, a refined characterization of variant-specific immune response for different polymorphic domains of PvMSP-1 is required. Here the association of allele-specific humoral immune responses and clinical parameters, as well as the association of variant-specific antibodies to exposure, parasitaemia and age among non-complicated *P. vivax* patients were assessed. These antibody responses were also analysed in relation to the haemoglobin concentration aiming to expand current knowledge of the PvMSP-1 variant-specific immune response in exposed populations.

## Methods

### Study population

The dataset analysed consisted of 141 non-complicated *P. vivax* mono-infected individuals randomly selected from a larger study described elsewhere [14]. Malaria infection was first diagnosed by microscopy examination of thick blood smears and then confirmed by PCR. Briefly, patients were recruited and treated in two different health centres in the Western Brazilian Amazon between February 2006 and January 2008: (1) 77 patients from the Hospital Universitário Júlio Müller in Cuiabá (Mato Grosso State), where active malaria transmission does not occur; (2) 64 patients from the Fundação de Medicina Tropical Dr. Heitor Vieira Dourado in Manaus (Amazonas State), located in a low malaria transmission area. Patients from Cuiabá reported short visits to other areas in the Brazilian endemic region suggesting that they had infected there. Patients from Cuiabá and Manaus were previously exposed to malaria as the median number of reported previous malaria episodes was two. All patients were examined and interviewed by a trained physician who filled in a questionnaire containing demographic, clinical, parasitological and epidemiological questions. Haemoglobin and platelet levels were measured using a blood cell counter (ABX Pentra 90; Horiba Diagnostics, Kyoto, Japan) and parasite densities were determined by examination of 200 fields at l000× magnification under oil immersion. Written informed consent was obtained from all study participants prior to sample collection and ethics approval was obtained from the Ethical Committee Research of Universidade Federal de Minas Gerais (Protocol # ETIC 060/07).

### Recombinant proteins

Eleven recombinant proteins corresponding to polymorphic regions (blocks 2 and 10) and conserved portions (block 13) of the PvMSP-1 [9] were used in this study. Five versions of block 2 (isolates Belem, BP29, BP39, BR07 and BP30), five versions of block 10 (isolates BP29, BP39, BP13, BR07 and BP01), and one version of the conserved block 13 (isolate Belem) were selected for the expression of recombinant antigens as previously described [13]. The 11 plasmids used here were kindly provided by Dr. Marcelo U. Ferreira (Universidade de São Paulo, Brazil) and all recombinant proteins were expressed in fusion to the C-terminus of *Schistosoma japonicum* glutathione S-transferase protein (GST). Briefly, PCR products were cloned into the pCR 2.1-TOPO vector (Invitrogen, Carlsbad, CA, USA) and subcloned into the expression vector pGEX-3X (Amersham Pharmacia Biotech). Recombinant proteins were expressed in transformed *Escherichia coli* (BL21-DE3) and purified by affinity chromatography as described elsewhere [13, 15]. Fractions were analysed by 12% SDS-PAGE and stained with Coomassie blue. Fractions containing recombinant proteins with a high degree of purity were pooled and extensively dialyzed against PBS. The protein concentration was determined using the BCA kit (Pierce, USA) according to the manufacturer’s recommendations.

#### Enzyme-linked immunosorbent assay (ELISA)

ELISA for total IgG antibodies was performed as described elsewhere with some modifications [14]. Briefly, flat-bottomed 96-well microtiter plates (Maxisorb; NUNC 4-39454; Denmark) were coated with 25 ng of recombinant antigens or GST (control antigen) in 50 µl of bicarbonate-carbonate buffer (pH 9.6; 0.1 M) and incubated overnight at 4 °C. After four washes with 0.05% Tween 20 in PBS, the wells were blocked by the addition of 5% (wt/vol) non-fat powdered milk in PBS for 120 min at 37 °C. Plasma samples diluted 1:160 (50 µl/well) were added following incubation at 37 °C for 90 min. A peroxidase-conjugated anti-human IgG (SIGMA, St Louis, MO, USA), diluted 1:2000, was added to each well and incubated at 37 °C for 90 min. Finally, o-phenylenediamine (SIGMA, USA) diluted in citrate–phosphate buffer (pH 5.0) containing hydrogen peroxide (50 μl/well) was added to detect monoclonal antibody binding. Fifteen minutes later, the reaction was stopped with 50 µl of H_2_SO_4_ 4N and the optical density (OD) was measured at 490 nm using an ELISA reader (BIO-RAD Lab USA). Each sample was assayed in duplicate and antibody values were expressed as reactivity index (RI), which was calculated as the ratio between the mean OD generated by each duplicate and the mean OD plus three standard deviations of samples from 20 Brazilian malaria-naïve blood donors never exposed to malaria. RIs equal or greater than 1.0 were scored as positive.

### PvMSP-1 gene amplification and sequencing

The block 10 polymorphic domain of the *PvMSP*-*1* gene was amplified and sequenced according to protocols previously described [13]. The primers PVF7 (5-CCTTAAGAATACCGAGATTTTGCTGAAG-3 [nucleotides 3429–3456]) and PVR3 (5-GCGATTACTTTGTCGTAG-3 [nucleotides 4007–3990]) were used. The sequences were analysed using the programs SEQUENCE SCANNER v1.0 (http://www.appliedbiosystems.com) and Bioedit v.7.0.5.3 [16]. Forty-one recovered sequences were aligned to other sequences previously described [9, 13] using CLUSTAL W following the identification and determination of haplotypes (alleles) distribution.

### Statistical analyses

The comparison of both sites, Cuiabá and Manaus, in terms of demographic, parasitological and laboratorial data was performed using either *T* test, Mann–Whitney test or Pearson’s Chi square test where appropriate. The McNemar test for paired samples was used to compare seroprevalence between pairs of antigens. The Wilcoxon signed-rank test for paired samples was used to compare the median antibody levels between pair of antigens. Pairwise correlation between levels of antibodies (reactivity indexes) against different antigens were calculated using Spearman’s non-parametric coefficients. Seroprevalence was estimated using a threshold of 1 for the reactivity indexes and the 95% Wilson confidence interval for proportions.

To test the association of each IgG response with haemoglobin concentration and platelet counts, a multiple linear regression approach was applied to the corresponding data considering the latter as the response variables. In particular, a null regression model for each response variable that included only the main effects of putative confounders (gender, age, study site, parasitaemia, and number of previous malaria exposure) was compared to an alternative model that included the same confounding effects plus the effect of the specific IgG response under testing. For the corresponding model comparison, likelihood ratio tests were performed in the corresponding data, where −log_10_(P value) was used as a measure of the strength of association (i.e., large values of −log_10_(P value) indicated a strong association between haemoglobin concentration and a given IgG response under testing). To control for multiple testing, the threshold for statistically significant was determined by the Bonferroni correction method [i.e., −log_10_ (overall significance level/number of tests performed)]. A similar approach was applied to analyse the association between anaemia (haemoglobin <11 g/dl) or thrombocytopaenia (platelet <150,000/mm^3^) and each IgG response but alternatively using a logistic regression framework due to binary nature of the response variables. To assess the quality of prediction of the fitted models, the area under the receiver-operating characteristic curve (AUC) was calculated for the models providing the strongest association between anaemia and IgG responses; the values of 0.5 and 1 correspond to a random and perfect prediction of the response variable by the model, respectively. In particular, high values of AUC indicated a good agreement between model predictions and the respective data.

All analysis were carried out in the R software version 3.3.0 for Apple Maverick operating system (http://www.r-project.org) using the authors’ own scripts, which are available from the authors upon request. The significance level was set at 5% for the analyses.

## Results

### Levels and prevalence of antibodies to polymorphic and conserved domains of PvMSP-1

Patients presenting non-complicated vivax malaria diagnosed in two different health centres (Cuiabá and Manaus) in the Brazilian Amazon region were enrolled in this study. The median parasitaemia significantly varied between patients from Cuiabá and Manaus (1640 vs. 3713 parasites/µl of blood, respectively; P < 0.0001). However, the groups are reasonably matched for age, gender, number of previous malaria episodes, haemoglobin levels, platelets count and number of white blood cells (Table 1).

**Table 1:** Demographic, parasitological and laboratorial data of the 141 patients infected by *Plasmodium vivax*

Characteristic	Median values (interquartile range)		P value
	Cuiabá (n = 77)	Manaus (n = 64)	
Age (years)	39 (26–48)	38 (27–49)	0.91
Gender (male %)	76.6	67.1	0.26
Number of previous episodes [n (%)]			0.07
0	16 (20.8)	19 (29.7)	
1–5	38 (49.4)	36 (56.2)	
>5	23 (29.9)	9 (14.1)	
Parasitaemia (parasites/µl)	1640.0 (686.5–2855.0)	3713.0 (1838.0–6733.0)	*<0.0001*
Haemoglobin (g/dl)	13.20 (11.50–14.30)	13.00 (12.15–14.50)	0.63
Platelets (cells/mm^3^)	107,000 (75,000–154,500)	100,000 (73,000–157,750)	0.54
White blood cells (cells/mm^3^)	5600 (4560–7250)	5000 (4300–6675)	0.06

Significant P values are indicated in italics

**Fig. 1:**
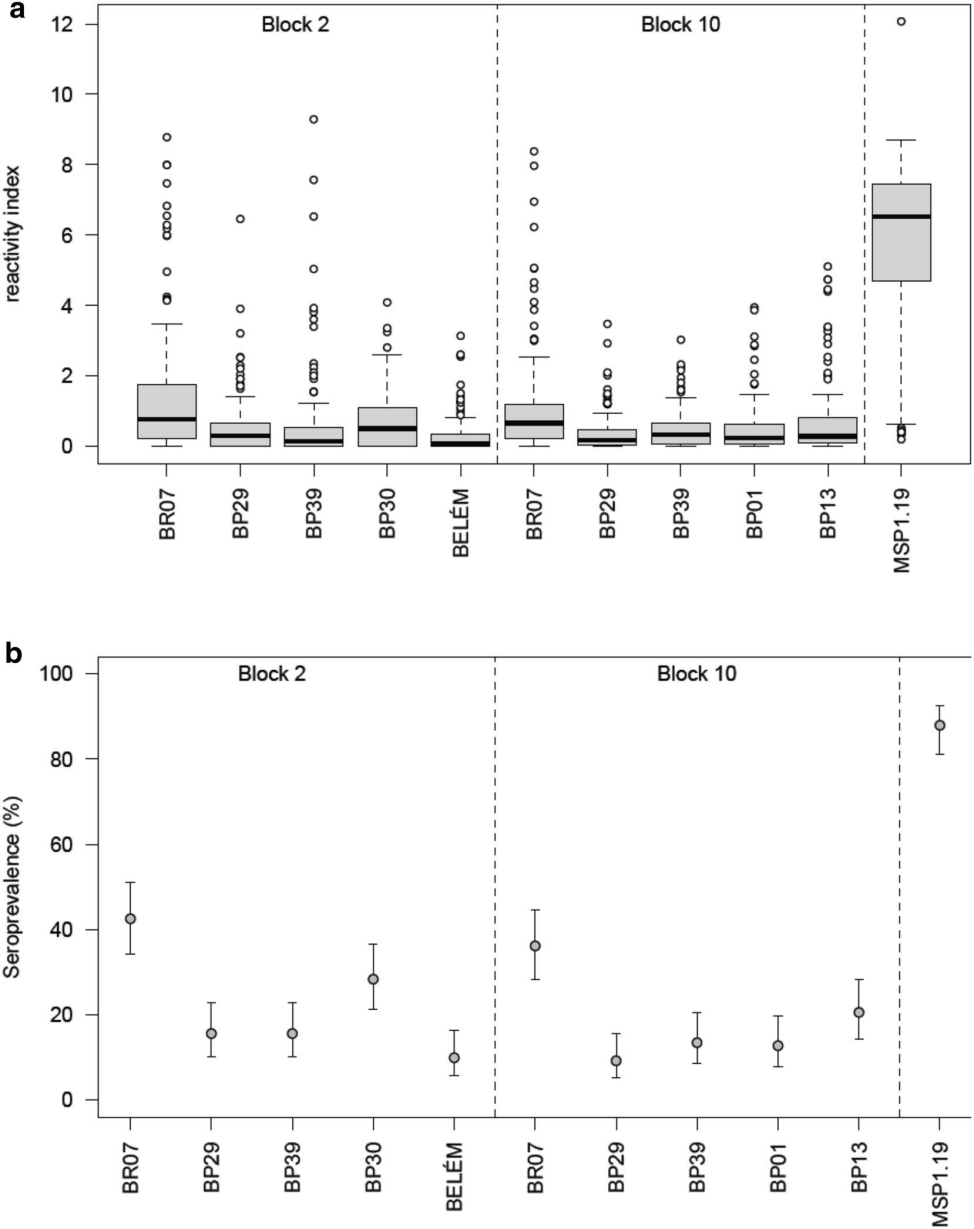
Antibody data for *P. vivax* patients. **a** Box plots of reactivity index of IgG antibodies to variable (block 2 and block 10) and conserved (block 13) recombinant antigens of PvMSP-1. Antibody reactivity of PvMSP-1_19_ antigen is statistically higher than that of each variant PvMSP-1 antigen (Wilcoxon signed-rank test, all P values <0.001). **b** Prevalence of seropositivity to each IgG responses and respective 95% confidence intervals, where seropositivity was determined as the reactivity index greater than 1. Seroprevalence of PvMSP-1_19_ antigen is statistically higher than that for each variant PvMSP-1 antigen (McNemar test, all P values <0.001)

Levels (measured as reactivity indexes) and seroprevalence of plasma IgG antibodies to 10 antigens representing allelic forms of PvMSP-1 (BR07, BP29, BP39, BP30 and BEL of block 2 and BR07, BP29, BP39, BP01 and BP13 of block 10) and to one conserved antigen representing block 13 (PvMSP-1_19_) are showed in Fig. 1a and b, respectively. As expected, the levels of IgG antibodies to PvMSP-1_19_ were significantly higher than those observed to other polymorphic antigens representing block 2 or block 10 of PvMSP-1 (all P values <0.001) (Fig. 1a). In close agreement with this observation is the high seroprevalence of patients with antibodies against PvMSP-1_19_ (87.9%) that contrasts with the low seroprevalence of the remaining antigens representing the variable region of the PvMSP-1 molecule. Among those, version BR07 of both block 2 and block 10 were the most recognized variable antigens (42.6 and 36.9%, respectively) (Fig. 1b). The other recombinant variable antigens were poorly recognized by *P. vivax* patients revealing seroprevalence no greater than 30%. Similar patterns were observed for plasma IgG levels confirming the poor antigenicity of variable antigens of PvMSP-1 as compared to conserved block 13, PvMSP-1_19_ (Fig. 1a).

### Antibody responses to polymorphic blocks of PvMSP-1 and their association with infecting parasites

**Fig. 2:**
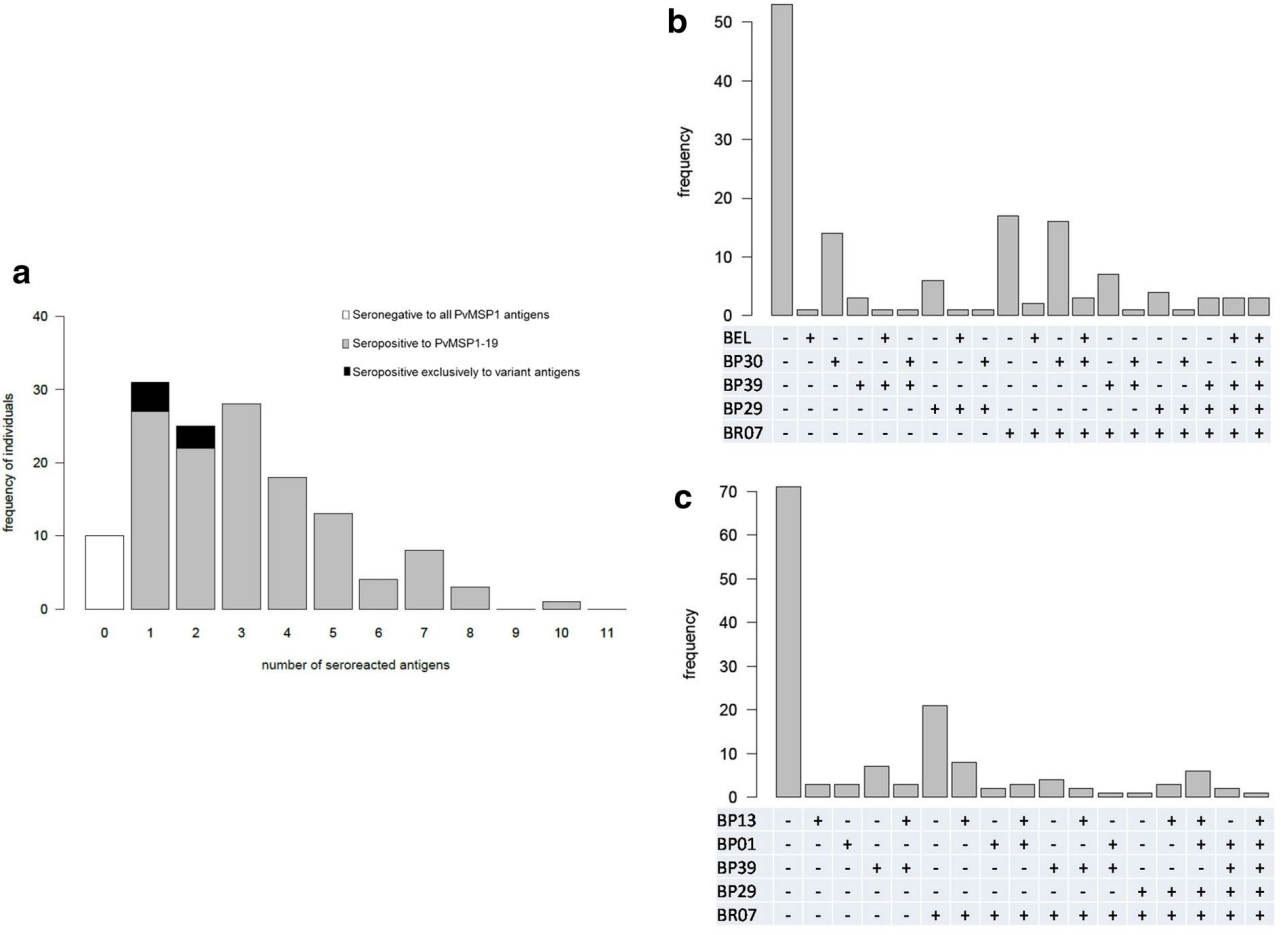
Frequencies of allele-specific IgG responses to PvMSP-1. **a** Frequency distribution of the total number of seropositive responses to conserved (PvMSP-1_19_) and variant antigens PvMSP-1 antigens. **b** Frequency distribution of different joint seropositivity profiles for block 2 variant antigens: BELÉM (BEL), BP30, BP39, BP29, and BR07 antigens. **c** Frequency distribution of different joint seropositivity profiles for block 10 variant antigens: BP13, BP01, BP39, BP29, and BR07 antigens

Ten out of 141 patients had no detectable IgG response to any antigen of the PvMSP-1 despite their current clinical infection (Fig. 2a). For PvMSP-1 responders, the proportion of patients who seroreacted to the conserved C-terminal PvMSP-1_19_ antigen was significantly higher than the proportion of patients that seroreacted to any variant antigen of block 2 or block 10. To assess the contribution of PvMSP-1_19_ determining such high antibody recognition rate, the range of positive responses (against one to 11 antigens tested) among 141 patients with positive IgG responses were analysed. As shown in Fig. 2a, the frequencies of seropositive patients who presented antibodies exclusively to any variant antigens were very low. Further, the overall combined frequency of antibody responses to all block 2 and block 10 variant antigens was significantly lower than that to C-terminal PvMSP-1_19_, a result that suggests a high natural diversity of past and current *P. vivax* infections. In line with this observation is the increased total number of seropositive antigens per individual as a function of the self-reported number of previous malaria cases. This high diversity of IgG response profiles is also captured by the combined seroprevalence profiles to block 2 and block 10 recombinant antigens shown in Fig. 2b and c, respectively.

Sequences of the *PvMSP*-*1* gene were obtained from a total number of 41 infected patients. These 41 sequences comprised eight different haplotypes from the block 10 *PvMSP*-*1* gene (see Additional file 1: Figure S1). Interestingly, version BR07, an isolate firstly collected in Porto Velho, the capital of Rondonia state (Western Brazilian Amazonia) in 1995, was the most common sequence among those 41 typed isolates (32%). The amino acid sequences of five haplotypes were identical to those of the antigens used for antibody measurements and were detected in 30 local variant sequences suggesting an adequate panel of antigens used for serology. Four sequences identified as type II, III, VI and VIII which present 100% of similarity to sequences of recombinant antigens BP13, BP29, BP01 and BP39 were recovered at frequencies of 7.3, 14.6, 9.8 and 9.8%, respectively (see Additional file 2: Table S1). Note that 11 isolates (28.6%) share less than 75% of amino acid identity with sequences of the antigens used but had homology with other antigen sequences: haplotypes V and VII showed similarities of up to 90% with Asian isolates and haplotype IV showed a similarity rate of 100% with another Brazilian isolated, BP30 not tested in our study.

### Weak associations between levels of IgG antibodies and variants belonging to the same block of PvMSP-1

**Fig. 3:**
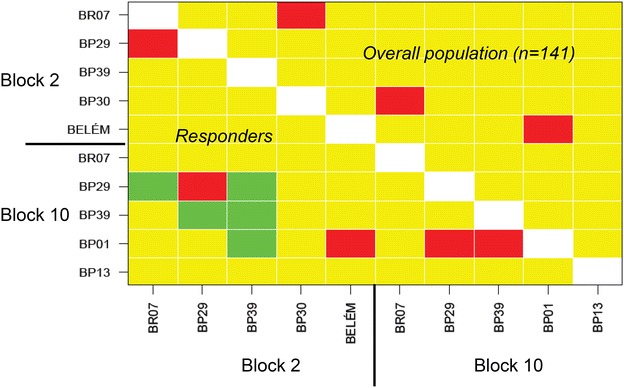
Pairwise correlation based on Spearman’s non-parametric coefficient between levels of IgG antibodies (reactivity index) to variant recombinant antigens of block 2 and block 10 of PvMSP-1 among overall population (n = 141 patients) and among responders to specific proteins (*green* correlation <−0.5, *yellow*—(−0.5) ≤ correlation ≤0.5, *red*—correlation >0.5)

A correlation analysis was performed between a pair of IgG antibodies against to 10 variant recombinant proteins among either the overall population (141 patients) or IgG responders for each pair of antigens simultaneously recognized. Most of the correlations provided evidence for weak associations between IgG levels to variants antigens belonging to the same block of PvMSP-1. Cross-reactivity between versions BR07 and both BR29 and BP30 of the block 2 and BP01 and both BP29 and BP39 of the block 10 were detected among IgG responders (Fig. 3). In contrast, those antigenic pairs of block 2 (BR29-BR07) and block 10 (BP01-BP29 and BP01-BP39) showed a moderate proportion of amino acid identity (69.6–77.1%) as previously reported [13]. Interestingly, inter-block positive correlations could be verified either among the overall population or IgG responders. For example, levels of IgG to BELEM version of the block 2 were moderately correlated to IgG levels to BP01 version of block 10. The same pattern was observed for the correlation between BP29 versions from either block 2 and block 10. Negative correlations were also obtained for inter antigenic blocks revealing five putative block 2–block 10 antigens pairs among responders (Fig. 3).

### Correlation between antibodies to PvMSP-1 and anaemia

**Fig. 4:**
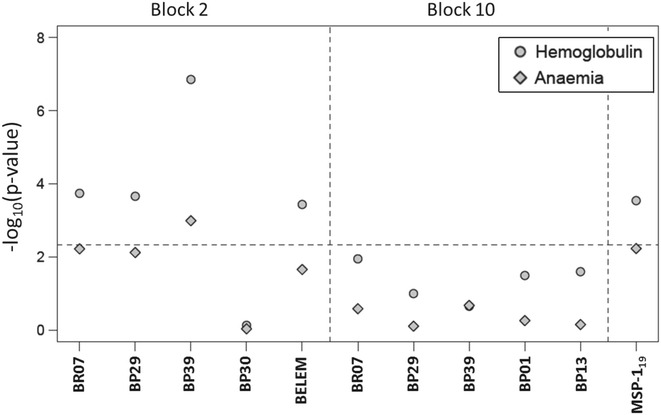
Associations between each IgG level and haemoglobin concentration (linear regression analysis) and anaemia (logistic regression model) where −log_10_(P value) is a measure of the strength of association and the *dashed line* refers to the threshold ensuring a 5% global statistical significance using the Bonferroni multiple testing correction

Multiple linear and logistic regression models were then used to determine whether platelets counts, haemoglobin levels or anaemia could be associated with each IgG antibody response to PvMSP-1 adjusting for putative confounding effects (i.e., locality, age, gender, parasitaemia and a total number of previous malaria episodes). Haemoglobin levels were associated with IgG antibody responses to not only the C-terminal of PvMSP-1 domain PvMSP-1_19_ but also with four among five variant antigens representing block 2 region (BR07, BP29, BP39, and BEL). The strongest association observed with haemoglobin levels refers to the IgG response to block 2 variant antigens, BP39. The respective association would appear to be negative as suggested by the negative estimate for antibody-level coefficient for the respective data (Table 2). None of the antibody responses to five antigens of the variable block 10 were associated with the underlying haemoglobin levels (Fig. 4). With respect to anaemia, there is only a strong association with IgG response to block 2 variant antigens, BP39. In close agreement with the haemoglobin data, this IgG response would appear to increase the odds of anaemia (Table 2). Finally, data suggests no strong association between platelet counts or thrombocytopaenia and any IgG response.

**Table 2:** Association analysis between antibodies against BP39 version of block 2 of PvMSP-1 and haemoglobin concentration (linear regression) and anaemia (logistic regression)

Coefficient	Haemoglobin concentration			Anaemia		
	Estimate	SE	P value	Estimate	SE	P value
Intercept	15.70	62.69	<0.001	−5.835	1.521	<0.001
Age (in years)	−0.015	0.010	0.164	0.035	0.023	0.131
Gender	−1.557	0.313	<0.001	2.198	0.676	0.001
Malaria episodes						
1–5	0.231	0.354	0.516	−1.222	0.711	0.086
>5	0.447	0.425	0.296	−1.825	0.966	0.059
Municipality (Manaus)	0.720	0.311	0.022	−2.501	0.894	0.005
Parasitemia (per 1000 parasites)	−0.111	0.039	0.005	0.205	0.918	0.025
Antibody level	−0.481	0.105	<10^−5^	0.629	0.220	0.004

## Discussion

MSP-1 has been considered one of the most promising molecules to be included in an anti-malarial subunit vaccine. However, its high allelic diversity observed in distinct *Plasmodium* isolates [16] might be one of the factors contributing to unsatisfactory results in the subsequent vaccine development. In fact, antigenic polymorphisms displayed by malaria parasites are considered as one of the strategies employed by them to escape from the host immune responses after recurrent infections [17]. Here, naturally acquired antibodies were studied in *P. vivax* infected patients from low and unstable malaria transmission settings. The results showed poor antigenicity of allelic variant antigens representing both block 2 and block 10 of PvMSP-1 in opposition to the high antigenicity of the conserved C-terminal, PvMSP-1_19_. The high magnitude of IgG response to the conserved C-terminal domain of the PvMSP-1 protein that is the only fragment that remains attached to the merozoite surface during erythrocyte invasion [18, 19] is an expected result and confirms previous data from several serological studies conducted in different epidemiological settings in the Brazilian endemic area [20–26].

A weak recognition of the antigens representing variable domains of PvMSP-1 was described in this study. This result is in contrast with a higher degree of recognition of variant proteins of blocks 2 and 10 (91.3 and 100% for at least one variant antigen of each block, respectively) among 27 subjects who were infected with *P. vivax* in a rural area in northwestern Brazil [13]. This contrasting evidence can be explained by several factors such as differences in the age of the patients, in the transmission level and geographical location and, in the stage of infection or in the degree of past exposure of the sampled individuals. In this study, the total number of ‘seropositive’ antigens per individuals increases with the self-reported number of previous malaria episodes. Similarly, an association between allelic-specific IgG responses to block 10 of PvMSP-1 antigens and the time of residence in the endemic Amazonian region was previously reported [13].

The seroprevalences concerning the BR07 version of both block 2 and block 10 were found to be higher as compared to other variant recognition. Indeed, this version was also found in relatively high frequency in infecting parasites based on sequencing of block 10 PvMSP-1 gene (32%). More importantly, there are 10 out of 13 (77%) patients carrying this specific haplotype that were seropositive against this recombinant protein. By contrast, Belem isolate has been previously reported as the predominant variant haplotype in a sample of *P. vivax* parasites living in the Manaus municipality (one of the same areas studied here) based on block 2 of PvMSP-1 sequencing [27]. In the present study, no sequencing of the block 2 of *PvMSP*-*1* gene was performed. The respective serological data suggests a low seroprevalence for such antigen but it is unclear how this seroprevalence relates to the frequency of parasites bearing this specific haplotype.

The results showed a weak antigenicity of block 2 and block 10 variable domains. Such weak response is unlikely to be explained by the restricted panel of recombinant proteins used here. In fact, the set of antigens used to measure IgG responses among *P. vivax* infected patients would appear to be appropriate since approximated 60% of the amino acid sequences recovered were identical to those of the antigens used and being detected in 30 out of 41 local variant sequences. The most likely explanation is the small contribution of each haplotype to the circulating population. However, this explanation remains to be confirmed with a higher number of sequenced *P. vivax* parasites and a larger set of samples from exposed individuals.

Association between the specificity of circulating antibodies and variants of *P. falciparum* infecting parasites has been extensively investigated in different endemic regions. No correlation between the allele-specific antibody response and variants of MSP-1 or MSP-2 circulating parasites were described in populations living in the Brazilian Amazon endemic region [28, 29]. However, it is much less clear about the association between allelic specific antibodies and PvMSP-1 sequences found in circulating parasites. In fact, only a single work has demonstrated frequent mismatches between PvMSP-1 sequences in infecting parasites and the antigenic variants recognized by IgG antibodies in the Brazilian Amazon endemic region [13].

Here, weak correlations between IgG responses were observed in pairs of variable antigens with high amino acid identity (>90%) contrasting data from that previous work conducted among 27 patients with acute *P. vivax* infection living in Acre state [13]. Therefore, the limited data available from PvMSP-1allele-specific antibodies undermined the possibility of discussing the deeper contribution of antigenic polymorphism on modulation of the naturally acquired immune response. In fact, even for vaccine based on PfMSP-1 which elicited antibodies that inhibited the growth of three diverse *P. falciparum* isolates in vitro [30] none protection against clinical malaria caused by diverse parasites in the field could be observed [31].

A dual role for specific antibodies against *P. falciparum* or *P. vivax* infection has been suggested for both immunity and pathogenesis of malaria (reviewed in [32]). Therefore, it was a reasonable hypothesis to investigate whether naturally acquired antibodies against conserved and variant PvMSP-1 proteins were associated with anaemia, a clinical parameter considered to be one of the major complications in *P. vivax* infections. The results suggested that none of the antibody responses to variable block 10 was associated with the haemoglobin levels of the sampled patients. However, haemoglobin levels were negatively correlated with IgG antibody responses to four variant antigens of block 2 domain (BR07, BP29, BP39, and BEL). These results are in agreement with a previous study that showed an association with an increased risk of infection and antibodies to block 2 of MSP-1 of *P. falciparum* [33].

The association between anaemia and serology specific has been suggested for antibodies against to *P. falciparum* rhoptry-associated proteins [34, 35]. In relation to *P. vivax*, parasitic antigens were detected on the surface of infected human RBCs [36] and an association between specific antibodies to PvMSP_3_ and anaemia has also been found [14]. It is possible to speculate that, during the proteolytic processing, PvMSP-1 polypeptides representing block 2 and block 10 domains could bind to the surface of infected or non-infected erythrocytes, form immune-complexes and stimulate phagocytosis or complement-mediated lysis. However, the precise mechanism underlying the association between anaemia and the role of specific antibodies determining such haematological disorder during malarial infection is still elusive and out of the scope of the present study.

Levels of IgG antibodies against PvMSP-1_19_ were found to be negatively associated with the haemoglobin levels. No statistically significant association was found between anaemia and the same antibodies. These two results might be explained by the IgG subclass distribution among serum samples from *P. vivax* patients. In a previous study on the same sample, anti-PvMSP-1_19_ non-cytophilic IgG2 and IgG4, as well as the cytophilic IgG3 antibodies, were detectable at similar proportions (approximately 50%) and levels [14]. Such non-cytophilic antibodies may block protective mechanisms such as parasite killing in cooperation with blood monocytes (ADCI) for *P. falciparum* [37]. This rationale may be then extended to explain the associations between antibodies to block 2 variant antigens and anaemia. Notwithstanding the fact that this study did not intend to characterise the subclass immune response to variable domains of PvMSP-1, it is yet possible that among patients infected by *P. vivax* who reported a short-term exposure to malaria transmission could present high proportions of non-cytophilic antibodies blocking any protective mechanism involved. In fact, block 2 domain of MSP-1 of *P. falciparum* has been considered an important target of ADCI mechanism [38]. However, the lack of an appropriate *P. vivax* culture method and the difficulties involved in performing in vivo assays using this species limit the tools available to test this hypothesis.

## Conclusions

Despite the limitations of the present study that undermined a deeper analysis towards the true causal relationships between allele-specific antibodies to block 2 of PvMSP-1 and anaemia, some of the conclusions are a step forward towards a better understanding of polymorphic domains as potential targets of vaccine development. Further studies are necessary to better understand the balance between the putative benefit of triggering an immune response and the possible deleterious effects of increasing the risk of anaemia.

## Additional files

**Additional file 1: Figure S1.** Alignment of sequences of the block 10 of PvMSP-1 found in parasites infecting 41 patients.

**Additional file 2: Table S1.** Haplotype frequencies among 41 *P. vivax* malaria patients and their associations with the block 10 polymorphic recombinant antigens used in the study.

## Abbreviations

MSP: merozoite surface protein
PvMSP-1: *Plasmodium vivax* merozoite surface protein 1
RBCs: red blood cells
PCR: polymerase chain reaction
ELISA: enzyme-linked immunosorbent assay
PBS: phosphate buffered saline
OD: optical density
RI: reactivity Index
AUC: receiver-operating characteristic curve
