# Supplementary material for: Allele-specific antibodies to Plasmodium vivax merozoite surface protein-1: prevalence and inverse relationship to haemoglobin levels during infection
Source: Malar J. 2016 Nov 16;15:559. doi: 10.1186/s12936-016-1612-z (PMC5112628; doi:10.1186/s12936-016-1612-z)
Supplement: Supplementary file 1 — Additional file 1: Figure S1. Alignment of sequences of the block 10 of PvMSP-1 found in parasites infecting 41 patients. [file 12936_2016_1612_MOESM1_ESM.pdf]

Alignment of sequences of the block 10 of PvMSP-1 found in parasites infecting 41 patients. Five sequences (I, II, III, VI and VIII corresponding to 73% of all sequences) are identical to the sequences of recombinant antigens used in serology (BR07, BP13, BP29, BP01, BP39, respectively). Sequences IV and V are quite similar to BP29 antigen (74.0% and 67.1% of amino acid similarity, respectively), whereas VII sequence has 63.7% of identity with BP01 antigen.

```
                  10        20        30        40        50        60        70        80        90
         ....|....|....|....|....|....|....|....|....|....|....|....|....|....|....|....|....|....|.
I        STSVA-----VTVPGAVVPG------VPTAAAAGSGASGAVP-------PATGPGAAA-----------------------GSTEENVAAK
II       STSVA-----VTVPGAVVPG------VPTAAAAGSGASGAVPPAGSV--PATGPGAAA-----------------------GSTEENVAAK
III      STSVA-----VTVPGAVVPG------VPTAAAAGSGASGAVPPAAAAGSGASGAVP--PAGGPSPPATGGVVPG-----VVESAEAQTKAQ
IV       STSVA-----VTVPGAVVPG------VPTAAAAGSGASGAVPPAGGPSPPAT----------------GGVVPG-----VVESAEAQTQTQ
V        STSVA-----VTVPGAVVPG------VPTAAAAGSGASGAVPPAGGPSPPAT----------------GGVVPGGVVPGVVESAEAQTQTQ
VI       GASAPAAAA-VTVPGAGVPAAAAVT-VPGAGVPAA--------GVVPGA-PA---GAA-PAGAAPAGAA---------PSAPGAQEQTQTQ
VII      GASAPAAAA-VTVPGAGVPAAGAGV-VPGAAAAGVVPGASAAGVVPGA-PA---GAA--------------------PSAPGAQEQTQTQ
VIII     GASTTAATLPVTVPSA-VPGGLPGAGVPGAAA-GLTPP---PPAGSV--PATGPGAAA-----------------------GSTEENVAAK
```
