# Supplementary material for: Allele-specific antibodies to Plasmodium vivax merozoite surface protein-1: prevalence and inverse relationship to haemoglobin levels during infection
Source: Malar J. 2016 Nov 16;15:559. doi: 10.1186/s12936-016-1612-z (PMC5112628; doi:10.1186/s12936-016-1612-z)
Supplement: Supplementary file 2 — Additional file 2: Table S1. Haplotype frequencies among 41 P. vivax malaria patients and their associations with the block 10 polymorphic recombinant antigens used in the study. [file 12936_2016_1612_MOESM2_ESM.docx]

**Table S1**

Haplotype frequencies among 41 *P. vivax* malaria patients and their associations with the block 10 polymorphic recombinant antigens used in the study

| **Haplotype** | **Corresponding antigen (% similarity)** | **Frequency** | **Seropositivity to corresponding antigen** |
| --- | --- | --- | --- |
| **I** | BR07 (100%) | 13(32%) | 10/13 (77%) |
| **II** | BP13 (100%) | 3 (7%) | 1/3 (33%) |
| **III** | BP29 (100%) | 6 (15%) | 4/6 (67%) |
| **IV** | BP29 (74%) | 3 (7%) | 1/3 (33%) |
| **V** | BP29 (67.1%) | 6 (15%) | 1/6 (17%) |
| **VI** | BP01 (100%) | 4 (10%) | 3/4 (75%) |
| **VII** | BP01 (63.7%) | 2 (5%) | 2/2 (100%) |
| **VII** | BP39 (100%) | 4 (10%) | 1/4 (25%) |
